# Efficacy of botanical lozenges in the treatment of chronic pharyngitis: a randomized controlled trial

**DOI:** 10.3389/fphar.2024.1162883

**Published:** 2024-03-14

**Authors:** Yi Wu, Feng Zhang, Dan Kuang, Dan Li, Jiai Yan, Ju Yang, Qinyue Wang, Yingyu Wang, Jing Sun, Yiran Liu, Yanping Xia, Hong Cao

**Affiliations:** ^1^ Nutritional Department, Affiliated Hospital of Jiangnan University, Wuxi, China; ^2^ Wuxi School of Medicine, Jiangnan University, Wuxi, China; ^3^ Clinical Assessment Center of Functional Food, Affiliated Hospital of Jiangnan University, Wuxi, China; ^4^ Yixing Institute of Food and Biotechnology Co., Ltd., Yixing, China; ^5^ Nursing Department, Affiliated Hospital of Jiangnan University, Wuxi, China; ^6^ Department of Endocrinology, Affiliated Hospital of Jiangnan University, Wuxi, China

**Keywords:** chronic pharyngitis, medicinal plants, pharyngeal symptoms and signs, illness perception, adherence to treatment

## Abstract

**Background:** In clinical practice, antibiotics and/or inhaled or oral hormone preparations are the first line of treatment for chronic pharyngitis. However, this therapeutic regimen is not satisfactory enough. At present, medicinal plants as dietary supplements or functional foods are widely recognized for the treatment and prevention of different diseases.

**Purpose:** This study aimed to evaluate the efficacy of the botanical lozenge made from several medicinal plant extracts in the treatment of chronic pharyngitis and its effects on patients’ illness perception and adherence to treatment.

**Methods:** Patients with chronic pharyngitis were randomly assigned to the experimental group (n = 52) or the control group (n = 51). Patients were given botanical lozenges prepared from the extracts of medicinal plants such as *Siraitia grosvenorii* (Swingle) C. Jeffrey ex A.M.Lu and Zhi Y. Zhang [Cucurbitaceae; Siraitiae fructus], *Lonicera japonica* Thunb [Caprifoliaceae; Lonicerae japonicae flos], *Platycodon grandiflorus* (Jacq.) A. DC [Campanulaceae; Platycodon radix], and *Glycyrrhiza uralensis* Fisch. ex DC [Fabaceae; Glycyrrhizae radix et rhizoma] or placebos made of starch for 15 days. The improvement of pharyngeal symptoms and signs, illness perception, and adherence to treatment were evaluated at the end of the intervention.

**Results:** The total score of pharyngeal symptoms of patients in the experimental group (3.33 ± 2.33) was significantly lower than that in the control group (5.20 ± 2.93) (*p* < 0.01). In comparison to the control group (3.43 ± 1.43), the total pharyngeal signs score of patients in the experimental group (2.69 ± 1.59) was considerably lower (*p <* 0.01). The improvement rates of pharyngeal itching, dry throat, pharyngeal foreign body sensation, aggravation due to excessive speaking, and congestion of pharyngeal mucosa in the experimental group were 73.81%, 67.50%, 67.57%, 65.22% and 44%, respectively, which were significantly higher than those in the control group (*p <* 0.05). In addition, patients taking botanical lozenges had better illness perception and adherence to treatment than those taking placebos (*p* < 0.05). Patients with low adherence to treatment showed less personal control, concerns, and understanding of chronic pharyngitis (*p* < 0.05).

**Conclusion:** Botanical lozenges not only aided patients in recovering from chronic pharyngitis but also improved their positive perceptions of the disease, which helped them adhere to their treatment regimen.

**Clinical Trial Registration**: [https://www.chictr.org.cn/], identifier [ChiCTR2200062139].

## 1 Introduction

Chronic pharyngitis (CP) is a chronic inflammation of the upper respiratory tract, involving the pharyngeal mucosa, submucosa, and lymphoid tissues (Li Z. et al., 2019). The clinical manifestations are mainly dry throat, sore throat, dry cough, or pharyngeal foreign body sensation (Ran et al., 2021). Currently, oral antibiotics are the first line of treatment for pharyngitis. When antibiotics are ineffective, oral or inhaled hormone preparations are added to treat pharyngitis (Ji et al., 2021). However, this therapeutic approach has some disadvantages, including its narrow therapeutic spectrum, high recurrence rate, and poor tolerance (Ji et al., 2021). Prolonged use of antibiotics can cause side effects, such as diarrhea, nausea, vomiting, rash, and drug resistance (van Driel et al., 2010). Long-term use of hormone preparations can lead to weight gain, hypertension, and osteoporosis (Hayward et al., 2017).

Some studies have found that some medicinal plants are effective in treating pharyngitis. For example, *Siraitia grosvenorii* (Swingle) C. Jeffrey ex A.M.Lu and Zhi Y. Zhang [Cucurbitaceae; Siraitiae fructus] is native to the China Southeast. It has been used as a botanical drug to treat pharyngeal pain and cough (Gong et al., 2019). As one of the main bioactive metabolites of Siraitia grosvenorii, Mogroside V has the function of regulating immunity (Duan et al., 2023). *Lonicera japonica* Thunb [Caprifoliaceae; Lonicerae japonicae flos] is a liana and native to China Southeast, Japan, Korea, Manchuria and Taiwan (Li et al., 2023). It is widely used to treat upper respiratory inflammation symptoms, such as cough and sore throat (Guo et al., 2021). One of its extracts, Chlorogenic acid, has anti-inflammatory, antibacterial, antioxidant, and other pharmacological effects (Yu et al., 2022). *Platycodon grandiflorus* (Jacq.) A. DC [Campanulaceae; Platycodon radix] has been widely used in Northeast Asia (including China, Japan, and Korea) to treat cough, excessive phlegm, and sore throat (Zhang et al., 2015). Its extract contains Glycosylated saponins and Platycodon D, which have been used as food health products for pulmonary diseases and respiratory disorders (Kang et al., 2019). *Glycyrrhiza uralensis* Fisch. ex DC [Fabaceae; Glycyrrhizae radix et rhizoma] is native to Asia and Southern Europe. It is used in the officinal medicine of Russia and included in the 14th edition of Russian Pharmacopoeia. It is also widely used in the European Union (Shikov et al., 2021). Besides helping to relieve cough, phlegm, and dyspnea, *G. uralensis* can reduce toxicity and increase the efficacy of certain medicinal plants when combined with them (Jiang et al., 2020). Some bioactive metabolites of *G. uralensis*, such as Liquiritin and Glycyrrhizic acid, have antioxidant, antiviral, anti-infective, and anti-inflammatory properties (Sharifi-Rad et al., 2021). In addition, these medicinal plants are also widely used as dietary supplements, daily foods, and functional foods to prevent and treat disease in many countries, such as China, Russia, Japan, Korea, Southeast Asia, South Africa, and South America (Shikov et al., 2017).

Although some pharmacologically active ingredients of medicinal plants are proven to be quite effective in treating diseases, their bitterness and odor tend to decrease patient compliance, compromising their curative potential in clinical applications (Zheng et al., 2018). Studies suggest that patients with chronic diseases are more susceptible to negative feelings (Wierenga et al., 2017). Patients experiencing negative emotions perceive the severity of the disease more intensely, which affects their recovery and quality of life to some extent (Giuffrida et al., 2021). Therefore, adherence and illness perception of patients with chronic diseases deserve to be given more attention in clinical trials of medicinal plants.

In this randomized controlled clinical trial, the studied botanical lozenge was made from extracts of *S. grosvenorii*, *L. japonica*, *P. grandiflorus*, and *G. uralensis*. We combined botanical lozenges with health education to investigate their efficacy against CP and their effect on patient’s illness perception and adherence to treatment.

## 2 Materials and methods

### 2.1 Participants

A total of 103 patients with CP were enrolled in the study and randomly assigned to the experimental group (n = 52) or control group (n = 51). Participants were adults (18–65 years of age) with persistent (>3 months) pharyngeal symptoms (sore throat, pharyngeal itching, or dry throat) or pharyngeal signs (pharyngeal edema, congestion of pharyngeal mucosa, or pharyngeal stasis of secretions). We excluded the following patients: serious diseases such as hematopoietic system, heart, brain, liver, and kidney; pregnant or lactating women; failure to adhere to treatment as prescribed; and taking antibiotics or other medications against pharyngitis during the intervention.

### 2.2 Study design and trial procedures

This study was a randomized and placebo-controlled trial. Patients with CP were randomly assigned to the experimental group or the control group according to the random number table that the research team made. Patients in the experimental group were given the botanical lozenge thrice daily for 15 days. Patients in the control group were given the matched placebo thrice daily for 15 days. Besides, patients in both groups received health education from the research team, including disease-related knowledge, medication care, and dietary care.

This study assessed patients at three-time points as follows: an inclusion visit (V1) on the first day, a telephone follow-up visit (V2) on the seventh day, and an end-of-intervention visit (V3) after 15 days. Patients and otolaryngologists did not know group allocation during the study.

At V1, an otolaryngologist determined the scores of patients’ pharyngeal symptoms and signs according to a 4-point scale. Pharyngeal symptoms had six indicators, including sore throat, pharyngeal itching, dry throat, dry cough, pharyngeal foreign body sensation, and aggravation due to excessive speaking. Pharyngeal signs had four indicators which included pharyngeal edema, congestion of pharyngeal mucosa, lymphatic follicle hyperplasia in the posterior pharyngeal wall, and pharyngeal stasis of secretions. The scores of individual indicators were as follows: none = 0, mild = 1, moderate = 2, and severe = 3 (Müller et al., 2016). The research team recorded patients’ basic information and collected their blood and urine specimens. Then performed imageology examinations on them, such as electrocardiogram, B-ultrasound, and chest X-ray. After completing the clinical examination, patients needed to take botanical lozenges or placebos the research team distributed on this day. The dosage of the botanical lozenge or placebo was three times a day and two tablets each time. Did not advise to eat or drink within half an hour after taking botanical lozenges or placebos.

At V2, through telephone follow-up, the research team assessed the effectiveness of the intervention and patient compliance by asking patients how they felt after taking botanical lozenges or placebos. All patients received medication guidance and dietary guidance once more.

At V3, that same otolaryngologist again scored patients’ pharyngeal symptoms and signs. Patients’ blood and urine specimens were collected again for safety testing. Additionally, patients needed to fill out the Chinese version of the Brief Illness Perception Questionnaire (BIPQ) and the eight-item Morisky Medication Adherence Scale (MMAS-8).

### 2.3 Study products

The studied botanical lozenge was mainly made from extracts of *S. grosvenorii fruits*, *L. japonica buds*, *P. grandiflorus roots*, and *G. uralensis roots*. The placebo was made from starch and had the same appearance as the botanical lozenge. The formulations of botanical lozenges and placebos are listed in Table 1. Botanical lozenges and placebos were provided and quality controlled by Suzhou Langbang Nutrition Company (Supplementary Materials and Methods). The batch numbers of 10 batches of botanical lozenges were 20220801, 20220805, 20220809, 20220902, 20220908, 20221001, 20221008, 20221101, 20221104, and 20221107. Following the dosage of traditional botanical drugs specified in the Chinese Pharmacopoeia (Yin et al., 2022) and the dose range of botanical drugs in phytopharmacological research (Heinrich et al., 2020), each patient in this study took one botanical lozenge or one placebo orally three times a day for 15 days. Botanical lozenges or placebos were prepared according to a standard production process (Supplementary Materials and Methods). The method of ingredient identification of the botanical lozenge was described in the Supplementary Materials and Methods.

**TABLE 1:** The formulations of botanical lozenges or placebos.

Group	Study products	Formulations
Experimental	Botanical lozenges	Extract of *Siraitia grosvenorii fruits* (210 g), extract of *Lonicera japonica flos buds* (160 g), extract of *Platycodon grandiflorus roots* (130 g), extract of *Glycyrrhiza uralensis* *roots* (60 g),Dextrin, Orange fruit powder, mannitol, Film coating agent, Magnesium stearate, Menthol, and Sucralose
Control	Placebos	Starch

Note: The above is made of 1000 pieces, 1.2 g/piece.

### 2.4 Outcome measures

The improvement rates of patients’ pharyngeal symptoms and signs at the end of the intervention served as a primary outcome measure. For the six indicators of pharyngeal symptoms and the four indicators of pharyngeal signs, a reduction of at least one point in the score was valid. It was invalid that there was no change in score (Müller et al., 2016). The remission rate greater than or equal to 33.33% was efficient for total pharyngeal symptoms and signs. The remission rates of symptoms and signs were the ratio of the total score before treatment minus the total score after treatment to the total score before treatment (Xu et al., 2020).

The secondary outcome measure was illness perception and adherence to treatment of patients during the study, which were measured by BIPQ (Farhat et al., 2019) and MMAS-8, respectively (Janežič et al., 2017). The BIPQ contains nine items. Eight items are used to evaluate the cognitive and emotional representations of illness. It includes consequences of the disease on daily life, a timeline for disease duration, personal control and treatment control over the disease, severity of symptoms, concern for the disease, understanding of the disease, and the disease-caused emotional representation. Each item ranges from 0 to 10. Personal control, treatment control, and understanding of the disease are reverse scoring items. The last item is an open-ended question asking patients to list the three most important causal factors in their illness. A total score of BIPQ comes from the sum of the 8 items. A higher score indicates a severe disease perception, whereas a lower score indicates a positive disease perception (Farhat et al., 2019). The MMAS-8 contains seven questions with “yes” or “no” options and one question on a 5-point Likert scale. The MMAS-8 has a score from 0 to 8. Scoring 8 points, 6 to <8 points, and <6 points on the scale match with high, medium, and low adherence, respectively (Janežič et al., 2017). Additionally, we assessed the safety of the botanical lozenge and placebo through blood and urine specimens.

### 2.5 Sample size calculation and statistical analysis

Following a comparison of two sample rates for a completely random design, the following formula gave an estimate of the needed sample size: n = (Z_α_ + Z_β_)^2^ 2P (1 - P)/(P_1_ - P_2_)^2^. P_1_ and P_2_ are the improvement rates in the experimental group and the control group, respectively. P is the mean of P_1_ and P_2_. A previously published study against pharyngitis showed that patients’ pharyngeal improvement rates were 79.5% in the lozenge group and 44.8% in the placebo group (Dao et al., 2019). At a significance level (alpha) of 0.05 and power (1-beta) of 0.9, it was assumed that the improvement rate of 45% in the control group and 75% in the experimental group. Considering a realistic drop-out of 10% (experience-based), this study estimated that each group consisted of 50 patients.

Continuous and normally distributed variables were expressed by means with standard deviations in this study. Statistical analysis used the *t*-test or analysis of variance. It presented categorical variables in frequencies and percentages and analyzed the statistics with Chi-square or Fisher’s exact test. Non-normally distributed data were presented as median (range) and were analyzed using the Mann-Whitney *U*-test or Kruskal–Wallis H test. Differences were statistically significant when *p*-value <0.05. All the analyses used SPSS 25.0 (IBM, New York, NY).

### 2.6 Ethical approval and registration

This study design and conduction followed the World Medical Association’s Declaration of Helsinki guidance. It was approved by the medical ethics committee of the Affiliated Hospital of Jiangnan University (LS2022021). Also, it was registered at the Chinese Clinical Trials Registry (https://www.chictr.org.cn/ Identifier: ChiCTR2200062139). Before being included, all patients signed an informed consent form allowing the use of their data in this study.

## 3 Results

### 3.1 Analysis of the major ingredients and fingerprint of botanical lozenges

The 50% methanolic extract of botanical lozenge was analyzed by UHPLC-HRMS/MS together with CD software. The typical total ion chromatogram scans of botanical lozenges in positive ion mode and negative ion mode are shown in Supplementary Figure S1. A total of 66 chemical ingredients were detected in the botanical lozenge. The formula and molecular weight are shown in Supplementary Table S1. It is worth noting that the important quality marker substances of *S. grosvenorii*, *L. japonica*, *P. grandiflorus*, and *G. uralensis* according to China Pharmacopoeia standard (Committee, 2015) were detected, such as Mogroside V, Chlorogenic acid, Platycodon D, and Liquiritin.

**FIGURE 1:**
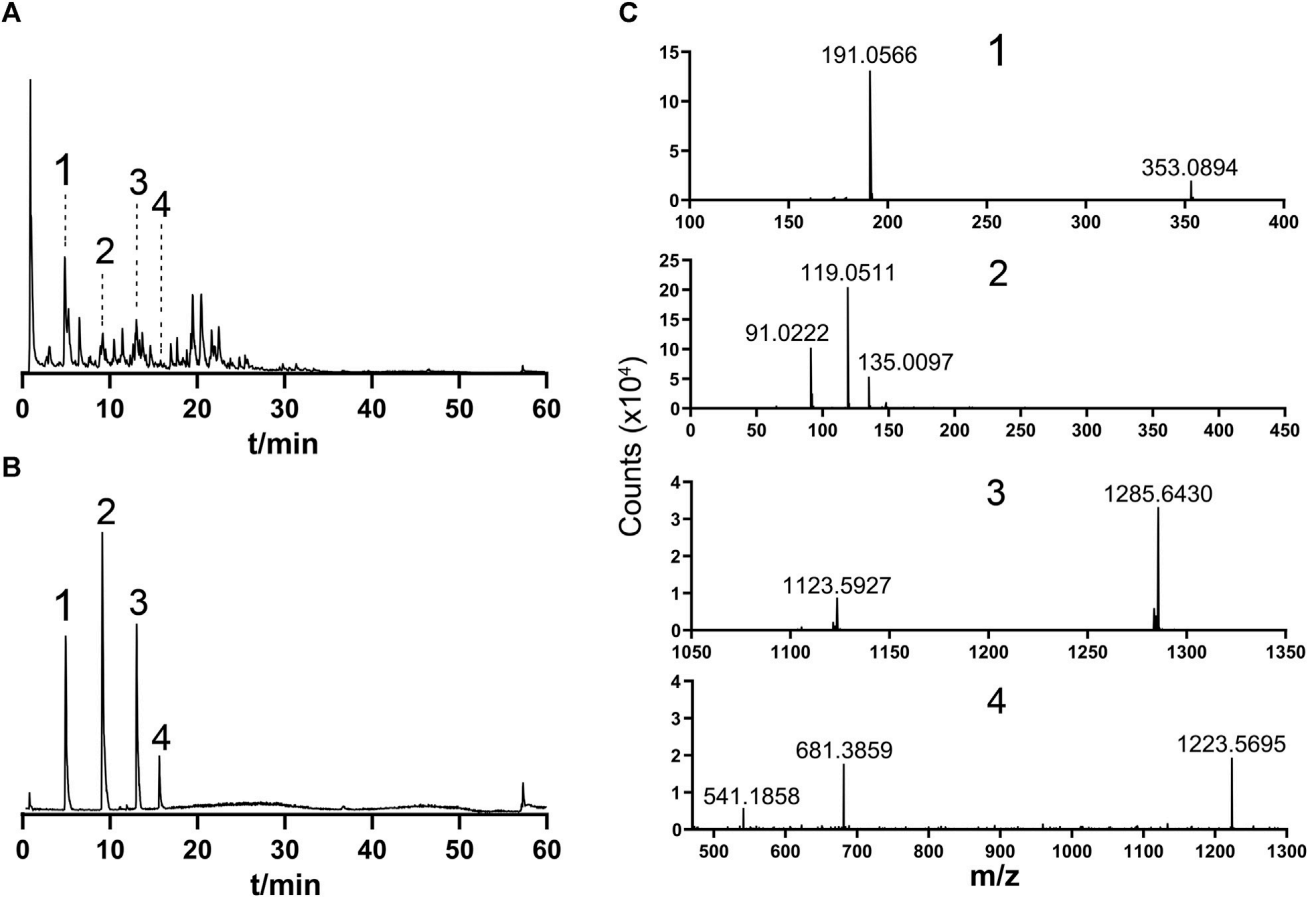
Fingerprint and secondary mass spectrometry. 1, Chlorogenic acid; 2, Liquiritin; 3, Mogroside V; 4, Platycodin D. **(A)** Typical LC/MS TIC fingerprint of botanical lozenges. **(B)** TIC chromatogram of standard samples. **(C)** MS^2^ spectra of 1, 2, 3, and 4 in Supplementary Figure S2. Precursor ions m/z are: 1, 353.0894; 2, 417.1191; 3, 1285.6430; 4, 1223.5695.

To standardize the fingerprint, 10 batches of botanical lozenges were analyzed. Peaks that existed in all 10 batches of botanical lozenges were assigned as “common peaks”. There were 18 “common peaks” in the TIC chromatogram (Supplementary Figure S2). From MS^2^ data, coupled with standard chromatogram, we have deduced Mogroside V, Chlorogenic acid, Platycodon D, and Liquiritin in the common peaks. The LC/MS fingerprint chromatogram of botanical lozenge was shown in Figure 1A, the chromatogram of mixture standard metabolites was shown in Figure 1B, and the MS^2^ spectra of peak numbers 1-4 were shown in Figure 1C.

### 3.2 Study population

**FIGURE 2:**
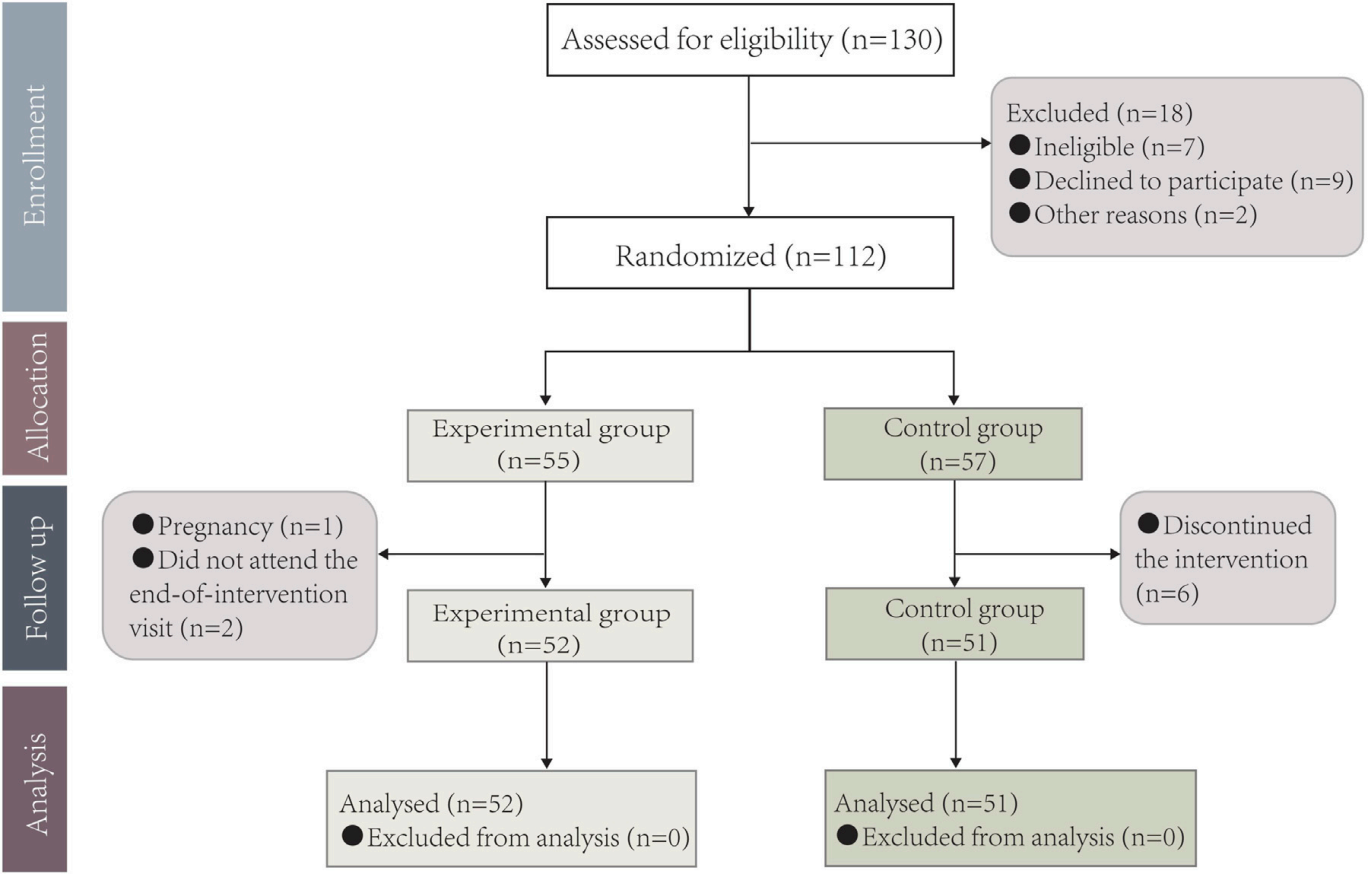
Participant flow diagram.

A total of130 patients were initially screened for eligibility and 18 patients were excluded. 112 patients with CP were randomly assigned to the experimental group (n = 55) or control group (n = 57). Nine patients dropped out during the study (two patients did not complete the end-of-intervention visit and one patient got pregnant in the experimental group and six patients discontinued the intervention in the control group). 103 patients (92.0%) completed this study for statistical analysis (Figure 2).

The two groups were well balanced in baseline demographics, health indicators (underlying diseases, poor eating habits, and sleep situation), and clinical characteristics (impact of CP on daily life, the total score of pharyngeal symptoms, and the total score of pharyngeal signs). The characteristics of the two groups in baseline were similar (*p* > 0.05) (Table 2). In addition, the chest X-ray, electrocardiogram, and abdominal B-ultrasound examination of two groups of patients did not show an obvious abnormality before the intervention.

**TABLE 2:** Demographic and clinical characteristics of enrolled patients.

Index		Experimental group (n = 52)	Control group (n = 51)	*p*-*value*
Gender	Male	17 (32.7%)	17 (32.3%)	0.945
	Female	35 (67.3%)	34 (66.7%)	
Age	Mean ± SD	39.38 ± 10.49	38.06 ± 9.03	0.494
Underlying diseases	No	42 (80.8%)	47 (92.2%)	0.149
	Yes	10 (19.2%)	4 (7.8%)	
Poor eating habits	No	36 (69.2%)	32 (62.7%)	0.537
	Yes	16 (30.8%)	19 (37.3%)	
Sleep situation	good	37 (71.2%)	33 (64.7%)	0.814
	general	13 (25%)	16 (31.4%)	
	poor	2 (3.8%)	2 (3.9%)	
Impact of CP on daily life	No	9 (17.3%)	8 (15.7%)	0.442
	Mild	27 (51.9%)	34 (66.7%)	
	Moderate	14 (26.9%)	8 (15.7%)	
	Severe	2 (3.9%)	1 (1.9%)	
Total score of pharyngeal symptoms	Mean ± SD	7.71 ± 3.34	7.12 ± 3.19	0.358
Total score of pharyngeal signs	Mean ± SD	4.06 ± 1.78	3.88 ± 1.58	0.598

### 3.3 Efficacy

#### 3.3.1 Improvement in pharyngeal symptoms

According to the assessments of otolaryngologists, the total scores of patients’ pharyngeal symptoms significantly decreased after the intervention in both the experimental group (*p <* 0.001) and the control group (*p <* 0.001) compared to the baseline. The total score of pharyngeal symptoms of patients in the experimental group (3.33 ± 2.33) was significantly lower than that those in the control group (5.20 ± 2.93) (*p <* 0.01) (Table 3).

**TABLE 3:** Total scores of pharyngeal symptoms and signs among the two groups before and after the intervention.

Group	Pharyngeal symptoms			Pharyngeal signs		
	Pre	Post	*p*-*value*	Pre	Post	*p*-*value*
Experimental	7.71 ± 3.34	3.33 ± 2.33***	0.001	4.06 ± 1.78	2.69 ± 1.59***	0.015
Control	7.12 ± 3.19	5.20 ± 2.93***		3.88 ± 1.58	3.43 ± 1.43**	

Note: *Compared with the group pre-intervention, *p <* 0.05; **Compared with the group pre-intervention, *p <* 0.01; ***Compared with the group pre-intervention, *p <* 0.001.

Patients taking botanical lozenges showed considerably higher improvement rates in pharyngeal symptoms than those taking placebos (*p <* 0.05). For single indicators of pharyngeal symptoms, the effective rates of pharyngeal itching, dry throat, pharyngeal foreign body sensation, and aggravation due to excessive speaking in the experimental group were 73.81, 67.50, 67.57, and 65.22%, respectively, which were significantly higher than those in the control group. However, compared to the experimental group, the effective rates of sore throat and dry cough in the control group were not statistically different (Table 4).

**TABLE 4:** Improvement of pharyngeal symptoms after intervention.

Symptoms	Group	Total	Effective number	Ineffective number	Effectiveness (%)	χ^2^	*p*-*value*
Sore throat	Experimental	27	22	5	81.48	2.572	0.109
	Control	32	20	12	62.5		
Pharyngeal itching	Experimental	42	31	11	73.81	3.872	*0.049*
	Control	38	20	18	52.63		
Dry throat	Experimental	40	27	13	67.50	5.488	*0.019*
	Control	43	18	25	41.86		
Dry cough	Experimental	37	28	9	75.68	3.272	0.070
	Control	31	17	14	54.84		
Pharyngeal foreign body sensation	Experimental	37	25	12	67.57	8.791	*0.003*
	Control	34	11	23	32.35		
Aggravation due to excessive speaking	Experimental	46	30	16	65.22	5.031	*0.025*
	Control	37	15	22	40.54		
Total	Experimental	52	39	13	75	6.380	*0.015*
	Control	51	26	25	50.98		

#### 3.3.2 Improvement in pharyngeal signs

After the intervention, there was a significant decrease in the total score of pharyngeal signs of patients in both the experimental group (*p <* 0.001) and the control group (*p <* 0.001). The total score of patients’ pharyngeal signs in the experimental group (2.69 ± 1.59) was significantly lower than those in the control group (3.43 ± 1.43) (*p <* 0.01) (Table 3).

Compared to the control group, patients in the experimental group had significantly higher improvement rates in pharyngeal signs (*p <* 0.05). Analysis of individual indicators of pharyngeal signs revealed that patients taking botanical lozenges had a higher effective rate of congestion of pharyngeal mucosa than those taking placebos (*p <* 0.05). The effective rate of lymphoid follicular hyperplasia in the posterior pharyngeal wall in the experimental group (44.68%) tended to be higher than that in the control group (26.09%) (*p* = 0.061). In addition, the effective rates of pharyngeal edema and pharyngeal stasis of secretions did not differ between the two groups (Table 5).

**TABLE 5:** Improvement of pharyngeal signs after intervention.

Signs	Group	Total	Effective number	Ineffective number	Effectiveness (%)	χ^2^	*p*-*value*
Pharyngeal edema	Experimental	23	13	10	56.52	2.710	0.100
	Control	27	9	18	33.33		
Congestion of pharyngeal mucosa	Experimental	50	22	28	44.00	6.297	*0.012*
	Control	49	10	39	20.41		
Lymphoid follicular hyperplasia in the posterior pharyngeal wall	Experimental	47	21	26	44.68	3.511	0.061
	Control	46	12	34	26.09		
Pharyngeal stasis of secretions	Experimental	16	7	9	43.75	−−	0.702
	Control	13	4	9	30.77		
Total	Experimental	52	28	24	53.85	6.321	*0.016*
	Control	51	15	16	29.41		

### 3.4 Illness perception and adherence to treatment of patients

The BIPQ score of the experimental group (38.46 ± 7.44) was significantly lower than that of the control group (41.46 ± 6.57) (*p <* 0.05). Further analysis of several items of the BIPQ revealed that there were significant differences in the timeline score (*p <* 0.01) and treatment control score (*p <* 0.01) between the two groups. Patients in the experimental group believed that their illness would last less time overall and that the therapy would be more effective than patients in the control group (Table 6). Compared to the control group, patients in the experimental group showed a higher rate of high and moderate adherence to treatment (*p <* 0.001). In the experimental group, about one-fifth of patients had low adherence to treatment, while in the control group about half of the patients had low adherence to treatment (Table 6).

**TABLE 6:** The scores of illness perception and adherence to treatment after intervention.

Index	Experimental group (n = 52)	Control group (n = 51)	Z/t	*p*-*value*
Dimensions of illness perceptions				
Consequences	4 (0–10)	5 (0–8)	−0.420	0.677
Timeline	5 (0–10)	6 (3–10)	−3.394	*0.001*
Personal control	5 (2–9)	5 (2–9)	−0.985	0.327
Treatment control	4 (0–7)	5 (1–10)	−2.587	*0.009*
Severity of symptoms	4 (1–8)	4 (2–9)	−0.205	0.839
Concerns	6 (0–9)	5 (1–10)	−0.400	0.692
Understanding	5 (0–10)	5 (0–10)	−1.048	0.297
Emotional representations	2 (0–10)	2 (0–10)	−0.380	0.707
BIPQ	38.65 ± 6.57	35.46 ± 7.44	2.302	*0.023*
Ranking of adherence to treatment			χ^2^	*p*-*value*
Low adherence	10 (19.2%)	30 (58.8%)	18.392	*0.000*
Medium adherence	30 (57.7%)	18 (35.3%)		
High adherence	12 (23.1%)	3 (5.9%)		

Note: Personal control, treatment control, and understanding were reverse-scoring items.

In addition, we assessed the level of illness perception among patients in the two groups with different adherence to treatment. In the experimental group, patients with low adherence had less personal control over their disease (*p <* 0.001) (Supplementary Table S2). Also, patients with low adherence tended to be less knowledgeable about the disease both in the experimental group (*p =* 0.061) and control group (*p =* 0.059) (Supplementary Table S2S). We also found that patients with low adherence to treatment in both groups had less personal control of their disease (*p <* 0.01) and understanding of the disease (*p <* 0.01), and were less concerned about the disease (*p <* 0.05) (Table 7).

**TABLE 7:** Illness perceptions of all patients with high, medium, and low adherence to treatment.

Dimensions of illness perceptions	Ranking of adherence to treatment			*p*-*value*
	Low (n = 40)	Medium (n = 48)	High (n = 15)	
Consequences	4 (1–10)	4 (0–8)	5 (1–9)	0.285
Timeline	6 (1–10)	5.5 (2–10)	5 (2–10)	0.109
Personal control	6 (2–9)	4 (2–8)	5 (4–9)	*0.002*
Treatment control	5 (1–8)	4 (0–10)	5 (0–7)	0.175
Severity of symptoms	4 (2–9)	4 (1–8)	4 (2–7)	0.953
Concerns	5 (1–10)	6 (1–10)	6 (0–8)	*0.026*
Understanding	6 (0–10)	5 (2–8)	5 (1–10)	*0.005*
Emotional representation	2 (1–10)	2 (0–8)	2 (0–10)	0.524
BIPQ	38.8 ± 6.73	35.6 ± 7.651	36.93 ± 5.982	0.114

Note: Personal control, treatment control, and understanding were reverse-scoring items.

### 3.5 Effects on human biochemical and other indicators

There were no significant differences in the levels of white blood cells (WBC), red blood cells (RBC), platelets (PLT), and hemoglobin (HGB) between the two groups of patients before and after the intervention. No significant differences in biochemical indicators of liver and kidney function such as total protein (TP), albumin (ALB), alanine transaminase (ALT), aspartate transaminase (AST), serum creatinine (SCR) between the two groups except Urea nitrogen (BUN). However, BUN levels in both the experimental and control groups were within the normal range (Supplementary Table S3).

## 4 Discussion

### 4.1 Efficacy evaluation

In this randomized and placebo-controlled clinical trial, we compared the efficacy of a botanical lozenge and a placebo in treating patients with CP. The botanical lozenge was significantly better than the placebo in improving pharyngeal symptoms and signs. The improvement of pharyngeal symptoms was more marked than that of pharyngeal signs in the short-term intervention.

The oral botanical lozenges used in this study were prepared from extracts of several medicinal plants that had therapeutic effects on pharyngitis. The extracts of *S. grosvenorii* and *L. japonica* make up the largest percentage of the formula of the botanical lozenges. They may play a large role in improving efficacy. According to some studies, the extract of *S. grosvenorii* (Mogroside V) can reduce the expression of inflammatory factors such as *IL-1*β, *IL -6*, and *TNF-*α, and inhibit the activation of *cox-2* (Shi et al., 2014; Li Y. et al., 2019). A study has shown that the botanical extract consisting of *S. grosvenorii*, *L. japonica*, and *broccoli seed* can relieve inflammation by downregulating the expression of inflammatory cytokines such as *IL-1*β, *IL-6*, *IL-8*, and *TNF-*α and affect the expression of tight junction proteins related to the integrity of gut epithelium (Jin et al., 2022).

In addition, this botanical extract produces other health benefits by favoring beneficial bacteria such as *Barnesiella* and *Akkermansia* (Jin et al., 2022). Expect *S. grosvenorii* and *L. japonica* flos, the remaining two medicinal plants are also helpful in improving the efficacy. The traditional pharmacological effect of *P. grandiflorum* is to reduce cough and expectorate (Ma et al., 2021). Its extract has anti-inflammatory effects by reducing lipopolysaccharide-induced inflammation (Si-Cong et al., 2021). It is known that *G. uralensis* has anti-inflammatory, anti-bacterial, antioxidative, anti-viral, and expectorant properties. The biologically active ingredients of *G. uralensis*, such as Glycyrrhizic acid and Liquiritin, inhibited the production of NO and inflammatory cytokine (Yu et al., 2015). *G. uralensis* extract was found to reduce colonization by *Streptococcus* mutans (Chen et al., 2019). Numerous studies have also shown that gut microbiota can usually participate in drug metabolism by producing specific enzymes, such as reductase and hydrolytic enzymes, to affect the efficacy, toxicity, and bioavailability of traditional botanical drugs (Xie et al., 2020). However, the specific regulatory mechanism remains unclear and needs further investigation.

Along with the obvious improvement of pharyngeal symptoms and signs in the experimental group, pharyngeal symptoms and signs were also relieved in the control group after the intervention, which indicated the existence of the placebo effect. Even though the placebo had a minimal therapeutic effect on pharyngitis, the total scores of pharyngeal symptoms and signs were decreased in the control group. In a study on the efficacy of thermal water nasal inhalation treatment for upper respiratory tract diseases, patients who practiced healthy behaviors, such as quitting smoking, increasing consumption of fruits and vegetables, and taking more dietary supplements had better treatment outcomes and compliance (Neri et al., 2018). In this study, more than one-third of patients in both groups had unhealthy eating habits at V1, including smoking, drinking, and eating spicy foods. During the intervention, patients of the two groups received disease-related health education and were largely aware of the risk factors for CP. As a result, they gradually adjusted their poor habits and eating patterns under our guidance. At V3, we found that patients in both groups had a moderate to high level of concern for the disease and understanding of the disease through the BIPQ. Therefore, we inferred that our health education improved patients’ understanding of the disease and affected their health behaviors, thereby helping to ease pharyngeal discomfort in the control group.

### 4.2 Illness perception and adherence to treatment of patients with pharyngitis

This study showed that patients in the experimental group had a better knowledge of the disease than patients in the control group. They believed that the illness would last less time and the botanical lozenges were more effective in relieving pharyngitis. A reported study indicated that illness perceptions can be changed (Chew et al., 2017), and indirectly influence the quality of life, functional recovery, and clinical parameters through adherence to treatment (Chew et al., 2017). Thus, our intervention might indirectly enhance clinical efficacy by elevating patient’s illness perceptions. In this study, patients taking the botanical lozenge had better adherence than those taking the placebo. A randomized controlled trial of viral pharyngitis revealed that the compliance of patients in the treatment group was significantly better than that in the control group (Dao et al., 2019). According to the follow-up on V2, patients thought the flavor of the botanical lozenge was noticeably superior to the taste of the placebo. They also claimed that the botanical lozenge cooled the throat. In general, medicines with good taste help to increase patient compliance and improve treatment efficacy. Besides comparing illness perception and adherence to treatment between the two groups, we also analyzed the level of illness perception in all patients with different adherence to treatment. We found that self-reported non-adherence was prevalent. More than one-third of patients were classified as having low adherence. A low level of adherence to treatment was related to lower personal control over the disease, less concern about the disease, and less understanding of the disease. A previous study identified illness perception as a significant predictor of self-reported adherence to treatment (Kim et al., 2021). Reinforcing patients’ positive perceptions and beliefs about the disease through personal strategies or medication treatment leads to better adherence to treatment, which might aid their recovery and disease management (Kim et al., 2021). Therefore, better treatment methods or interventions are needed to improve the illness perception of patients with chronic diseases to improve adherence to treatment and promote recovery of patients.

### 4.3 The advantages of botanical lozenges

In clinical practice, the first line of treatments for CP mainly includes antibiotics and/or inhaled or oral hormone preparations. Overuse of antibiotics often results in dysbiosis of the throat microbiota (Korkmaz et al., 2022), which leads to double infection and even further deterioration of the disease. Repeated aerosol inhalation therapy for CP is still ineffective (Li C. et al., 2019). Traditional medicinal plants are effective for treating pharyngitis, but the traditional decoction approach is somewhat laborious (Zhang et al., 2017). With the advancement of medical technology, the dosage forms of medicinal plants have become more and more diversified, including soup, pill, tablet, oral liquid, and tea (Zhang et al., 2017). Currently, many classic botanical formulas have been developed into Chinese patented drugs or functional foods. For example, *G. uralensis* oral solution used as a Chinese patented drug treats upper respiratory infections, bronchitis, colds, and coughs (Jiang et al., 2020). Botanical tea is used as a functional food to alleviate the symptoms in CP patients. Some botanical teas are mostly prepared from traditional botanical drugs, such as *L. japonica*, *G. uralensis*, and *P. grandiflorus* (Li C. et al., 2019). As a result, it is very convenient and effective for patients to use botanical lozenges in the treatment of CP. In China, many traditional botanical drugs are effective in the prevention and treatment of diseases. Several studies have revealed that the secondary metabolites of botanical drugshave antioxidative, antibacterial, antiviral, anticancer, and anti-inflammatory properties (Lu et al., 2022). As more and more people pay attention to their health, the acceptance of botanical drugs among the population is increasing. For instance, botanical tea is gaining popularity as one of the most enjoyable drinks due to its health-promoting benefits (Wijesundara and Rupasinghe, 2019). Compared with oral or inhaled hormone preparations, medicinal plants may be safer and more prevalent.

In our trial, the botanical lozenge was prepared from medicinal plants with edible properties. Manufacturers processed the extract of several medicinal plants into oral tablets. They had the advantages of convenience, economy, relative safety, and some efficacy. In summary, this safe, effective, and convenient botanical lozenge prepared from medicinal plants provided new options for preventing and treating CP.

### 4.4 Strengths and limitations

This is one of the few studies to examine the effectiveness of extracts from medicinal plants in the treatment of CP. Oral administration is widely used as an acceptable and relatively safe method of drug administration for patients. We made these medicinal plants into convenient and economical botanical lozenges. We also combined botanical lozenges with health education, such as disease-related knowledge, medication guidance, dietary guidance, and follow-up, to treat CP. Additionally, we investigated patients’ adherence to treatment, illness perception level, and therapeutic safety. Furthermore, we analyzed the relationship between adherence to treatment and illness perception of the included patients, which provided directions for future treatment and management of chronic diseases.

However, this study had some limitations. First, the patients in this study were recruited from a single institution, which might limit data generalization. Also, the assessment of pharyngeal symptoms and signs was relatively subjective. In the future, evaluating patients’ clinical outcomes needs more comprehensive evaluation methods and more precise objective measures (e.g., inflammatory factors).

## 5 Conclusion

The botanical lozenge prepared from the extracts of medicinal plants was effective in relieving the pharyngeal symptoms and signs of CP. The combination of these medicinal plants and health education not only improved patients’ positive illness perception but also helped them adhere to treatment regimens. In general, this study elucidated the therapeutic and health-promoting effects of the novel extracts of medicinal plants and health education on CP.

## Data Availability

The original contributions presented in the study are included in the article/Supplementary Material, further inquiries can be directed to the corresponding authors.

## References

[B1] Chen Y. Agnello M. Dinis M. Chien K. C. Wang J. Hu W. (2019). Lollipop containing Glycyrrhiza uralensis extract reduces Streptococcus mutans colonization and maintains oral microbial diversity in Chinese preschool children. PLoS One 14 (8), e0221756. 10.1371/journal.pone.0221756 31442287 PMC6707631

[B2] Chew B. H. Vos R. C. Heijmans M. Shariff-Ghazali S. Fernandez A. Rutten G. (2017). Validity and reliability of a Malay version of the brief illness perception questionnaire for patients with type 2 diabetes mellitus. BMC Med. Res. Methodol. 17 (1), 118. 10.1186/s12874-017-0394-5 28774271 PMC5543429

[B3] Dao V. A. Overhagen S. Bilstein A. Kolot C. Sonnemann U. Mösges R. (2019). Ectoine lozenges in the treatment of acute viral pharyngitis: a prospective, active-controlled clinical study. Eur. Arch. Otorhinolaryngol. 276 (3), 775–783. 10.1007/s00405-019-05324-9 30739176 PMC6411829

[B4] Duan J. Zhu D. Zheng X. Ju Y. Wang F. Sun Y. (2023). Siraitia grosvenorii (swingle) C. Jeffrey: research progress of its active components, pharmacological effects, and extraction methods. Foods 12 (7), 1373. 10.3390/foods12071373 37048193 PMC10093486

[B5] Farhat R. Assaf J. Jabbour H. Licha H. Hajj A. Hallit S. (2019). Adherence to oral glucose lowering drugs, quality of life, treatment satisfaction and illness perception: a cross-sectional study in patients with type 2 diabetes. Saudi Pharm. J. 27 (1), 126–132. 10.1016/j.jsps.2018.09.005 30662315 PMC6323195

[B6] Giuffrida S. Fiala S. Barro L. Pazzi S. Soldini E. Levati S. (2021). Description and analysis of disease representation in chronic patients through the Illness Perception Questionnaire (IPQ-r): implications for clinical practice. Prof. Inferm. 74 (4), 219–226. 10.7429/pi.2021.744226 35363957

[B7] Gong X. Chen N. Ren K. Jia J. Wei K. Zhang L. (2019). The fruits of Siraitia grosvenorii: a review of a Chinese food-medicine. Front. Pharmacol. 10, 1400. 10.3389/fphar.2019.01400 31849659 PMC6903776

[B8] Guo X. Yu X. Zheng B. Zhang L. Zhang F. Zhang Y. (2021). Network pharmacology-based identification of potential targets of Lonicerae japonicae flos acting on anti-inflammatory effects. Biomed. Res. Int. 2021, 5507003. 10.1155/2021/5507003 34595237 PMC8478540

[B9] Hayward G. N. Hay A. D. Moore M. V. Jawad S. Williams N. Voysey M. (2017). Effect of oral dexamethasone without immediate antibiotics vs placebo on acute sore throat in adults: a randomized clinical trial. Jama 317 (15), 1535–1543. 10.1001/jama.2017.3417 28418482 PMC5470351

[B10] Heinrich M. Appendino G. Efferth T. Fürst R. Izzo A. A. Kayser O. (2020). Best practice in research - overcoming common challenges in phytopharmacological research. J. Ethnopharmacol. 246, 112230. 10.1016/j.jep.2019.112230 31526860

[B11] Janežič A. Locatelli I. Kos M. (2017). Criterion validity of 8-item Morisky medication adherence scale in patients with asthma. PLoS One 12 (11), e0187835. 10.1371/journal.pone.0187835 29190693 PMC5708647

[B12] Ji S. Xu F. Zhu R. Wang C. Guo D. Jiang Y. (2021). Mechanism of yinqin oral liquid in the treatment of chronic pharyngitis based on network Pharmacology. Drug Des. Devel Ther. 15, 4413–4421. 10.2147/DDDT.S324139 PMC8542895 34707348

[B13] Jiang M. Zhao S. Yang S. Lin X. He X. Wei X. (2020). An "essential herbal medicine"-licorice: a review of phytochemicals and its effects in combination preparations. J. Ethnopharmacol. 249, 112439. 10.1016/j.jep.2019.112439 31811935

[B14] Jin L. Deng L. Bartlett M. Ren Y. Lu J. Chen Q. (2022). A novel herbal extract blend product prevents particulate matters-induced inflammation by improving gut microbiota and maintaining the integrity of the intestinal barrier. Nutrients 14 (10), 2010. 10.3390/nu14102010 35631153 PMC9145798

[B15] Kang S. H. Kim T. H. Shin K. C. Ko Y. J. Oh D. K. (2019). Biotransformation of food-derived saponins, platycosides, into deglucosylated saponins including deglucosylated platycodin D and their anti-inflammatory activities. J. Agric. Food Chem. 67 (5), 1470–1477. 10.1021/acs.jafc.8b06399 30652865

[B16] Kim H. Sereika S. M. Lingler J. H. Albert S. M. Bender C. M. (2021). Illness perceptions, self-efficacy, and self-reported medication adherence in persons aged 50 and older with type 2 diabetes. J. Cardiovasc Nurs. 36 (4), 312–328. 10.1097/JCN.0000000000000675 32304467 PMC7572490

[B17] Korkmaz H. Çetinkol Y. Korkmaz M. Çalgın M. K. Kaşko Arıcı Y. (2022). Effect of antibiotic exposure on upper respiratory tract bacterial flora. Med. Sci. Monit. 28, e934931. 10.12659/MSM.934931 34987147 PMC8750656

[B18] Li C. Wu F. Yuan W. Ding Q. Wang M. Zhang Q. (2019c). Systematic review of herbal tea (a traditional Chinese treatment method) in the therapy of chronic simple pharyngitis and preliminary exploration about its medication rules. Evid. Based Complement. Altern. Med. 2019, 9458676. 10.1155/2019/9458676 PMC6791273 31662783

[B19] Li J. Chang X. Huang Q. Liu P. Zhao X. Li F. (2023). Construction of SNP fingerprint and population genetic analysis of honeysuckle germplasm resources in China. Front. Plant Sci. 14, 1080691. 10.3389/fpls.2023.1080691 36938035 PMC10017979

[B20] Li Y. Zou L. Li T. Lai D. Wu Y. Qin S. (2019b). Mogroside V inhibits LPS-induced COX-2 expression/ROS production and overexpression of HO-1 by blocking phosphorylation of AKT1 in RAW264.7 cells. Acta Biochim. Biophys. Sin. (Shanghai). 51 (4), 365–374. 10.1093/abbs/gmz014 30877761

[B21] Li Z. Huang J. Hu Z. (2019a). Screening and diagnosis of chronic pharyngitis based on deep learning. Int. J. Environ. Res. Public Health 16 (10), 1688. 10.3390/ijerph16101688 31091759 PMC6572379

[B22] Lu Q. Li R. Yang Y. Zhang Y. Zhao Q. Li J. (2022). Ingredients with anti-inflammatory effect from medicine food homology plants. Food Chem. 368, 130610. 10.1016/j.foodchem.2021.130610 34419798

[B23] Ma X. Shao S. Xiao F. Zhang H. Zhang R. Wang M. (2021). Platycodon grandiflorum extract: chemical composition and whitening, antioxidant, and anti-inflammatory effects. RSC Adv. 11 (18), 10814–10826. 10.1039/d0ra09443a 35423572 PMC8695864

[B24] Müller D. Lindemann T. Shah-Hosseini K. Scherner O. Knop M. Bilstein A. (2016). Efficacy and tolerability of an ectoine mouth and throat spray compared with those of saline lozenges in the treatment of acute pharyngitis and/or laryngitis: a prospective, controlled, observational clinical trial. Eur. Arch. Otorhinolaryngol. 273 (9), 2591–2597. 10.1007/s00405-016-4060-z 27126336 PMC4974281

[B25] Neri M. Sansone L. Pietrasanta L. Kisialiou A. Cabano E. Martini M. (2018). Gene and protein expression of CXCR4 in adult and elderly patients with chronic rhinitis, pharyngitis or sinusitis undergoing thermal water nasal inhalations. Immun. Ageing 15, 10. 10.1186/s12979-018-0114-y 29497453 PMC5828426

[B26] Ran F. Han X. Deng X. Wu Z. Huang H. Qiu M. (2021). High or low temperature extraction, which is more conducive to Triphala against chronic pharyngitis? Biomed. Pharmacother. 140, 111787. 10.1016/j.biopha.2021.111787 34091181

[B27] Sharifi-Rad J. Quispe C. Herrera-Bravo J. Belén L. H. Kaur R. Kregiel D. (2021). Glycyrrhiza genus: enlightening phytochemical components for pharmacological and health-promoting abilities. Oxid. Med. Cell Longev. 2021, 7571132. 10.1155/2021/7571132 34349875 PMC8328722

[B28] Shi D. Zheng M. Wang Y. Liu C. Chen S. (2014). Protective effects and mechanisms of mogroside V on LPS-induced acute lung injury in mice. Pharm. Biol. 52 (6), 729–734. 10.3109/13880209.2013.867451 24621273

[B29] Shikov A. N. Narkevich I. A. Flisyuk E. V. Luzhanin V. G. Pozharitskaya O. N. (2021). Medicinal plants from the 14(th) edition of the Russian Pharmacopoeia, recent updates. J. Ethnopharmacol. 268, 113685. 10.1016/j.jep.2020.113685 33309919

[B30] Shikov A. N. Tsitsilin A. N. Pozharitskaya O. N. Makarov V. G. Heinrich M. (2017). Traditional and current food use of wild plants listed in the Russian Pharmacopoeia. Front. Pharmacol. 8, 841. 10.3389/fphar.2017.00841 29209213 PMC5702350

[B31] Si-Cong L. Chaoqin R. Ge L. Xu-Ting L. Jin-Liang L. Bin W. (2021). Platycodon grandiflorum extract attenuates lipopolysaccharide-induced acute lung injury via TLR4/NF-κBp65 pathway in rats. Pak J. Pharm. Sci. 34 (6), 2213–2218. 35034883

[B32] van Driel M. L. De Sutter A. I. Keber N. Habraken H. Christiaens T. (2010). Different antibiotic treatments for group A streptococcal pharyngitis. Cochrane Database Syst. Rev. 10, Cd004406. 10.1002/14651858.CD004406.pub3 20927734

[B33] Wierenga K. L. Lehto R. H. Given B. (2017). Emotion regulation in chronic disease populations: an integrative review. Res. Theory Nurs. Pract. 31 (3), 247–271. 10.1891/1541-6577.31.3.247 28793948 PMC5992894

[B34] Wijesundara N. M. Rupasinghe H. P. V. (2019). Herbal tea for the management of pharyngitis: inhibition of Streptococcus pyogenes growth and biofilm formation by herbal infusions. Biomedicines 7 (3), 63. 10.3390/biomedicines7030063 31450579 PMC6783935

[B35] Xie Y. Hu F. Xiang D. Lu H. Li W. Zhao A. (2020). The metabolic effect of gut microbiota on drugs. Drug Metab. Rev. 52 (1), 139–156. 10.1080/03602532.2020.1718691 32116054

[B36] Xu C. Yue R. Lv X. Wu T. Yang M. Chen Y. (2020). The efficacy and safety of Banxia-Houpo-Tang for chronic pharyngitis: a protocol for systematic review and meta analysis. Med. Baltim. 99 (30), e19922. 10.1097/MD.0000000000019922 PMC7387004 32791655

[B37] Yin X. B. Qu C. H. Dong X. X. Shen M. R. Ni J. (2022). Preparation regularity of Chinese patent medicine in Chinese Pharmacopoeia (2020 edition, Vol.Ⅰ). Zhongguo Zhong yao za zhi 47 (16), 4529–4535. 10.19540/j.cnki.cjcmm.20220419.601 36046882

[B38] Yu H. Guo K. Lai K. Shah M. A. Xu Z. Cui N. (2022). Chromosome-scale genome assembly of an important medicinal plant honeysuckle. Sci. Data 9 (1), 226. 10.1038/s41597-022-01385-4 35610245 PMC9130202

[B39] Yu J. Y. Ha J. Y. Kim K. M. Jung Y. S. Jung J. C. Oh S. (2015). Anti-Inflammatory activities of licorice extract and its active compounds, glycyrrhizic acid, liquiritin and liquiritigenin, in BV2 cells and mice liver. Molecules 20 (7), 13041–13054. 10.3390/molecules200713041 26205049 PMC6332102

[B40] Zhang L. Wang Y. Yang D. Zhang C. Zhang N. Li M. (2015). Platycodon grandiflorus - an ethnopharmacological, phytochemical and pharmacological review. J. Ethnopharmacol. 164, 147–161. 10.1016/j.jep.2015.01.052 25666431

[B41] Zhang X. Xie Y. M. Li G. X. Gao Y. Zhao Y. C. Tang J. J. (2017). Advantages and problems of traditional Chinese medicine in treatment of acute pharyngitis. Zhongguo Zhong Yao Za Zhi 42 (19), 3819–3825. 10.19540/j.cnki.cjcmm.20170901.002 29235301

[B42] Zheng X. Wu F. Hong Y. Shen L. Lin X. Feng Y. (2018). Developments in taste-masking techniques for traditional Chinese medicines. Pharmaceutics 10 (3), 157. 10.3390/pharmaceutics10030157 30213035 PMC6161181

