# Supplementary material for: Efficacy of botanical lozenges in the treatment of chronic pharyngitis: a randomized controlled trial
Source: Front Pharmacol. 2024 Mar 14;15:1162883. doi: 10.3389/fphar.2024.1162883 (PMC10973001; doi:10.3389/fphar.2024.1162883)
Supplement: Supplementary file 1 [file Table1.docx]

Supplementary Material

Efficacy of botanical lozenges in the treatment of chronic pharyngitis: a randomized controlled trial

Yi Wu *, Feng Zhang, Dan Kuang

*** Correspondence:** Hong Cao: HongCao@jiangnan.edu.cn

# Supplementary Materials and Methods

## 1.1 Quality control of study products

Botanical lozenges and placebos used in this study were from the same place. Their main chemicals were determined by the Wuxi Institute for Drug Control. According to the determination method recorded in the “China Pharmacopoeia (2015 edition)” and National Food Safety Standards, the botanical drugs conformed to meet the China Pharmacopoeia standard (Committee, 2015) and National Food Safety Standards.

## 1.2 The standard production process of the botanical lozenge

Firstly, *Siraitia grosvenorii fruits*, *Lonicera japonica buds*, *Platycodon grandiflorus* *roots*, and *Glycyrrhiza uralensis roots* were cut into small pieces and crushed before being weighed. Secondly, extracting (one alcohol extraction and two water extraction) and filtering the weighting formula. Thirdly, combining the above three filtrates before concentration. Fourthly, the concentrated liquid was heated to achieve sterilization. Fifthly, spray drying of sterilized concentrated liquid and collection of spray drying powder (i.e. mixed extract of *Siraitia grosvenorii fruits*, *Lonicera japonica buds*, *Platycodon grandiflorus* *roots*, and *Glycyrrhiza uralensis roots*). Sixthly, weighing the mixed extracts and other substances. Seventhly, sifting and mixing the weighted formula. Eighthly, pelletizing (Moisture content ≤ 4.0%) and sifting the whole grain (10-65 mesh particles). Tenthly, tableting (the range of weight was controlled to ± 5.0%, the range of hardness was 8~14kp, and the disintegration time ≤ 60 minutes) and film coating.

## 1.3 The standard production process of the placebo

Firstly, weighing starch. Secondly, sieving with an 80-mesh vibrating screen. Thirdly, pelletizing and sifting the whole grain (10-65 mesh particles). Fourthly, tableting (the range of weight was controlled to ± 5.0%, the range of hardness was 8~14kp, and the disintegration time ≤ 60 minutes) and film coating.

## 1.4 Identification of ingredients of the botanical lozenge

The chemical ingredients of botanical lozenges were identified using ultra-high-performance liquid chromatography-high resolution tandem mass spectrometry (UHPLC-HRMS/MS) together with the Compound Discoverer 3.1 (CD) software (1, 2). The samples were analyzed using the Vanquish UHPLC system and Q-Exactive Orbitrap mass spectrometer equipped with an electrospray ionization source (Thermo Scientific, USA). Chromatographic separation was carried out using a Hypersil GOLD C18 column (1.9 μm particle size, 100 × 2.1 mm, Thermo Scientific, USA) at a flow rate of 0.3 mL/min. The mobile phase was composed of 0.1% acetic acid in water (A) and CH_3_CN (B), and the elution gradient program was: 0–50 min, 5–95% B; 50–55 min, 95–95% B; 55–56 min, 95–5% B; and 56–60 min, 5–5% B. The mass spectrometry (MS) analysis was operated in both positive and negative ion modes (full MS/dd-MS2), its parameters were set as follows: spray voltage was 3.9 kV; capillary temperature was 320 °C; sheath air pressure was 40 kPa; collision energy was 10 and 40 eV; scanning range was M/z 100-1500; and both MS and MS^2^ resolutions were set to 70000. The compound identification was assigned by matching their tandem mass spectra with the ChemSpider and mzCloud mass spectral library in CD software.

For the fingerprint of botanical lozenges, the MS was changed to negative ion mode, and the total ion chromatogram (TIC) was used as LC/MS fingerprint (3, 4). To identify the peaks of the fingerprint, four standard samples (Chlorogenic acid, Liquiritin, Platycodin D, and Mogroside V) were purchased from the National Institutes for Food and Drug Control (Beijing, China). Furthermore, ion pairs of m/z 353→191 (for Chlorogenic acid) (5), m/z 417 →135/119/91 (for Liquiritin) (6), m/z 1223.6→681 (for Platycodin D) (7) and m/z 1,285.6 → 1,123.6 (for Mogroside V) (8) in the spectrometry were also used to identify peaks.

# Supplementary Figures and Tables

## 2.1 Supplementary Figures


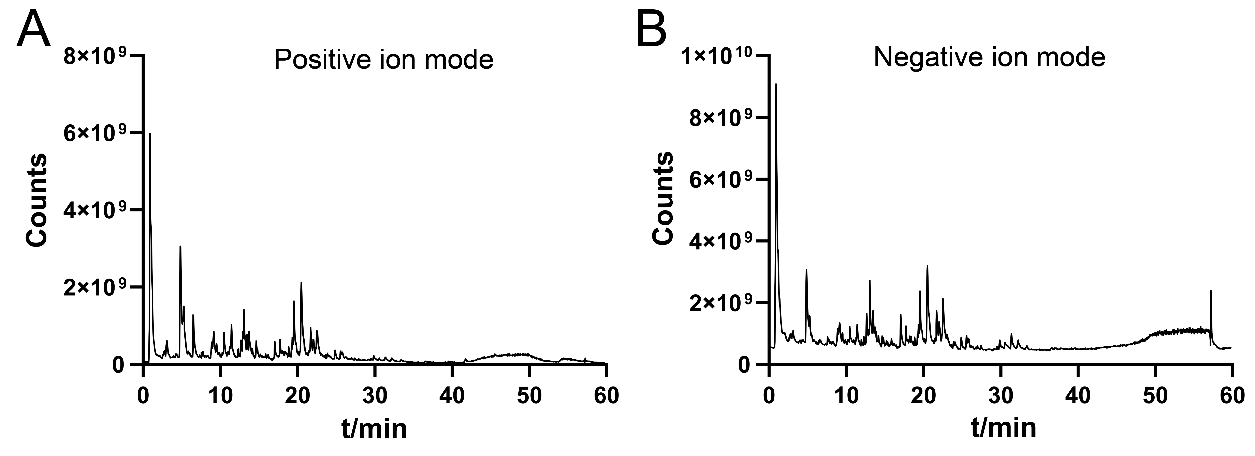


**Supplementary Figure S1** Total ion chromatogram of botanical lozenges in positive ion mode (A) and negative ion mode (B).


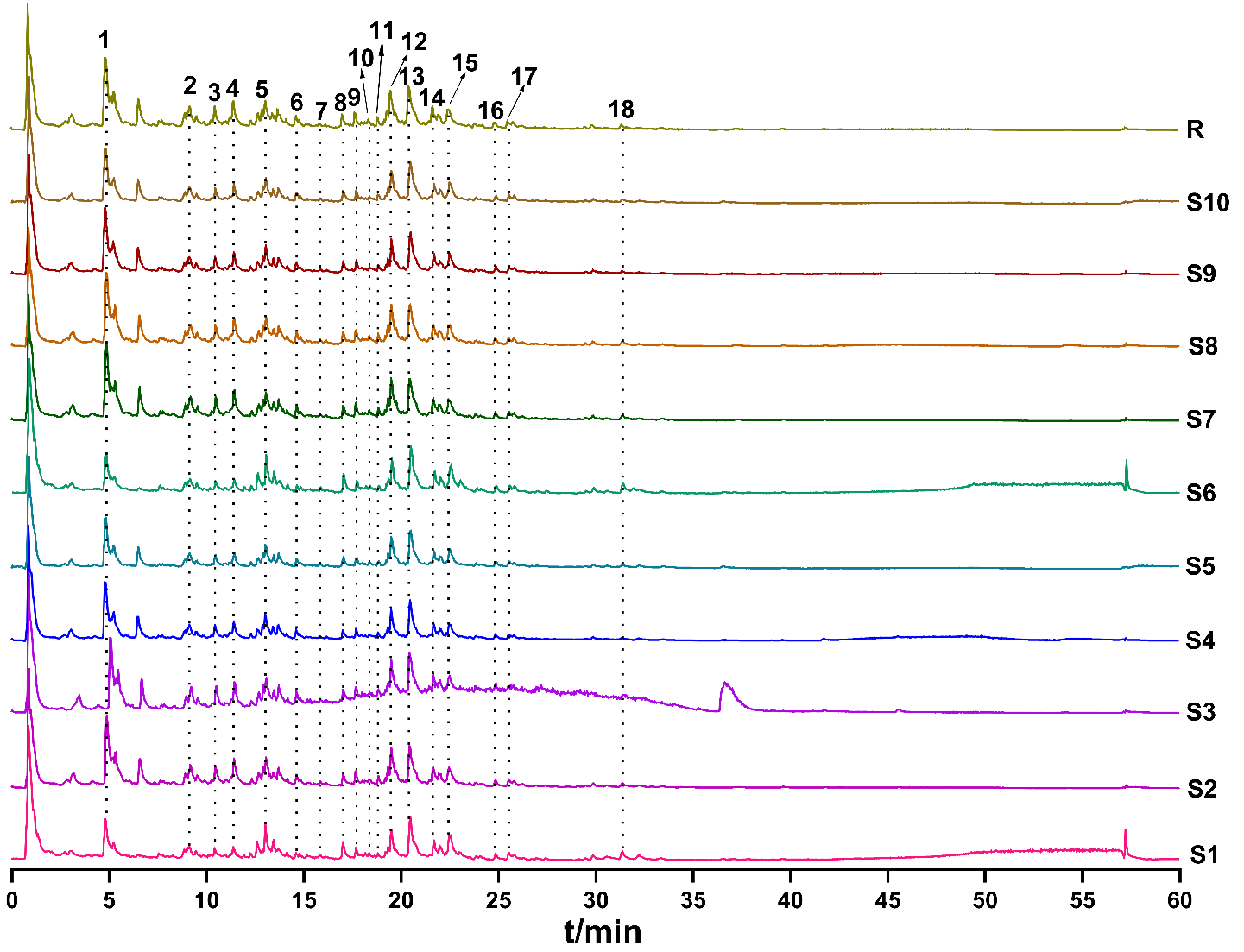


**Supplementary Figure S2** The HUPLC-ESI/MS fingerprint of 10 batches of botanical lozenges.

## 2.1 Supplementary Tables

**Supplementary Table S1** Chemical ingredients of botanical lozenges

| **Number** | **Name** | **Formula** | **Molecular Weight** | **Number** | **Name** | **Formula** | **Molecular Weight** |
| --- | --- | --- | --- | --- | --- | --- | --- |
| 1 | Genistein | C_15_H_10_O_5_ | 270.0525 | 35 | N-Acetyl-DL-tyrosine | C_11_H_13_NO_4_ | 223.0841 |
| 2 | Licuraside | C_26_H_30_O_13_ | 550.1678 | 36 | Pinoresinol Diglucoside | C_32_H_42_O_16_ | 682.2476 |
| 3 | 5-Oxo-L-prolyl-L-leucine | C_11_H_18_N_2_O_4_ | 242.1264 | 37 | Rotundic acid | C_30_H_48_O_5_ | 488.3502 |
| 4 | Liquiritin apioside | C_26_H_30_O_13_ | 550.1687 | 38 | Asiatic acid | C_30_H_48_O_5_ | 488.3500 |
| 5 | Myristyl sulfate | C_14_H_30_O_4_S | 294.1867 | 39 | Thapsic acid | C_16_H_30_O_4_ | 286.2144 |
| 6 | Azelaic acid | C_9_H_16_O_4_ | 188.1043 | 40 | Quinoa saponin 9 | C_42_H_66_O_15_ | 810.4408 |
| 7 | Isoliquiritin Apioside | C_26_H_30_O_13_ | 550.1687 | 41 | BMK glycidic acid | C_10_H_10_O_3_ | 178.0622 |
| 8 | Gentisic acid 5-O-Î²-glucoside | C_13_H_16_O_9_ | 316.0798 | 42 | CIMICIFUGOSIDE H-2 | C_35_H_54_O_10_ | 634.3729 |
| 9 | 4-Dodecylbenzenesulfonic acid | C_18_H_30_O_3_S | 326.1914 | 43 | Alpha-dimorphecolic acid | C_18_H_32_O_3_ | 296.2347 |
| 10 | Daidzein | C_15_H_10_O_4_ | 254.0577 | 44 | Mogroside V | C_60_H_102_O_29_ | 1286.6491 |
| 11 | Warfarin | C_19_H_16_O_4_ | 308.1048 | 45 | Nictoflorin | C_27_H_30_O_15_ | 594.1590 |
| 12 | 18-β-Glycyrrhetinic acid | C_30_H_46_O_4_ | 470.3391 | 46 | Ginsenoside-Rg2 | C_42_H_72_O_13_ | 784.4966 |
| 13 | Liquiritin | C_21_H_22_O_9_ | 418.1264 | 47 | 2,5-di-tert-Butylhydroquinone | C_14_H_22_O_2_ | 222.1616 |
| 14 | Soyasaponin I | C_48_H_78_O_18_ | 942.5194 | 48 | 2,5-Dihydroxycinnamic acid | C_9_H_8_O_4_ | 180.0414 |
| 15 | Platycodin D | C_57_H_92_O_28_ | 1224.5775 | 49 | Emmolic Acid | C_30_H_46_O_5_ | 486.3339 |
| 16 | 4-Hydroxyphenylpyruvate | C_9_H_8_O_4_ | 180.0414 | 50 | Apiin | C_26_H_28_O_14_ | 564.1485 |
| 17 | Licoagroside B | C_18_H_24_O_12_ | 432.1270 | 51 | Wogonin | C_16_H_12_O_5_ | 284.0687 |
| 18 | Stearic acid | C_18_H_36_O_2_ | 284.2711 | 52 | Narcissin | C_28_H_32_O_16_ | 624.1695 |
| 19 | Ginsenoside-Rg3 | C_42_H_72_O_13_ | 784.4961 | 53 | Quillaic Acid | C_30_H_46_O_5_ | 486.3343 |
| 20 | Linoleic acid | C_18_H_32_O_2_ | 280.2403 | 54 | Hydrastinine | C_11_H_13_NO_3_ | 207.0891 |
| 21 | Veronicastroside | C_27_H_30_O_15_ | 594.1590 | 55 | Chlorogenic acid | C_16_H_18_O_9_ | 354.0947 |
| 22 | D- (+) -Tryptophan | C_11_H_12_N_2_O_2_ | 204.0893 | 56 | Isoliquiritigenin | C_15_H_12_O_4_ | 256.0742 |
| 23 | Isorhamnetin 3-O-neohesperidoside | C_28_H_32_O_16_ | 624.1695 | 57 | (4E)-6-Hydroxy-4-octadecenoic acid | C_18_H_34_O_3_ | 298.2506 |
| 24 | 4-Methyl-2-oxo-2H-chromen-7-yl alpha-D-mannopyranoside | C_16_H_18_O_8_ | 338.1001 | 58 | Hydrangenol 8-O-beta-D-glucopyranoside | C_21_H_22_O_9_ | 418.1265 |
| 25 | Coumarin, 4,5,7-trihydroxy-3-(p-methoxyphenyl)- | C_16_H_12_O_6_ | 300.0635 | 59 | 10-Hydroxy-12-octadecenoic acid | C_18_H_34_O_3_ | 298.2506 |
| 26 | 2,2'-Methylenebis(4-methyl-6-tert-butylphenol) | C_23_H_32_O_2_ | 340.2406 | 60 | 2-(Î²-D-Glucopyranosyloxy)-5-hydroxybenzoic acid | C_13_H_16_O_9_ | 316.0798 |
| 27 | 7-Hydroxy-2',2'-dimethyl-8-(3-methyl-2-buten-1-yl)-2,3-dihydro-2'H,4H-2,6'-bichromen-4-one | C_25_H_26_O_4_ | 390.1832 | 61 | (24E)-3-[(2-O-Hexopyranosylhexopyranosyl)oxy]-22,26-dihydroxy-9,19-cyclolanost-24-en-28-oic acid | C_42_H_68_O_15_ | 812.4580 |
| 28 | 2-[(1S,2S,4aR,8aS)-1-hydroxy-4a-methyl-8-methylidene-decahydronaphthalen-2-yl]prop-2-enoic acid | C_15_H_22_O_3_ | 250.1570 | 62 | 1-O-[(3beta)-3-(beta-L-Glucopyranuronosyloxy)-23-hydroxy-28-oxoolean-12-en-28-yl]-beta-D-glucopyranose | C_42_H_66_O_15_ | 810.4408 |
| 29 | 1,3,5-trihydroxy-4-{[(2E)-3-(3-hydroxy-4-methoxyphenyl)prop-2-enoyl]oxy}cyclohexane-1-carboxylic acid | C_17_H_20_O_9_ | 368.1110 | 63 | 1-Octen-3-yl 6-O-[(2R,3R,4R)-3,4-dihydroxy-4-(hydroxymethyl)tetrahydro-2-furanyl]-beta-D-glucopyranoside | C_19_H_34_O_10_ | 422.2154 |
| 30 | 4-(3,4-dihydroxyphenyl)-7-hydroxy-5-{[(2S,3R,4S,5S,6R)-3,4,5-trihydroxy-6-(hydroxymethyl)oxan-2-yl]oxy}-2H-chromen-2-one | C_21_H_20_O_11_ | 448.1008 | 64 | (1S)-1,5-Anhydro-1-[7-hydroxy-3-(4-hydroxy-3-methoxyphenyl)-4-oxo-4H-chromen-8-yl]-D-erythro-hexitol | C_22_H_22_O_10_ | 446.1218 |
| 31 | Aurantioobtusin | C_17_H_14_O_7_ | 330.0741 | 65 | 4-[(1R,3aS,4R,6aS)-3a,6a-Dihydroxy-4-(4-hydroxy-3-methoxyphenyl)tetrahydro-1H,3H-furo[3,4-c]furan-1-yl]-2-methoxyphenyl beta-D-glucopyranoside | C_26_H_32_O_13_ | 552.1853 |
| 32 | 5,7-Dihydroxy-2-(4-hydroxyphenyl)-4-oxo-4H-chromen-3-yl 2-O-(6-deoxy-beta-D-glucopyranosyl)-alpha-L-mannopyranoside | C_27_H_30_O_15_ | 594.1580 |  |  |  |  |
| 33 | 1,3,4-Trihydroxy-5-{[(2E)-3-(4-hydroxyphenyl)-2-propenoyl]oxy}cyclohexanecarboxylic acid | C_16_H_18_O_8_ | 338.1001 | 66 | 3-O-[(2R,3R,4R)-3,4-Dihydroxy-4-(hydroxymethyl)tetrahydro-2-furanyl]-beta-D-xylopyranosyl-(1->4)-6-deoxy-alpha-L-mannopyranosyl-(1->2)-1-O-[(2beta,3beta,5xi,6beta,9xi,16alpha)-3-(beta-D-glucopyranuron osyloxy)-2,6,16,23-tetrahydroxy-28-oxoolean-12-en-28-yl]-alpha-L-arabinofuranose | C_57_H_90_O_29_ | 1238.5582 |
| 34 | 7-{[4,5-dihydroxy-6-(hydroxymethyl)-3-{[(2S,3R,4R,5R,6S)-3,4,5-trihydroxy-6-methyloxan-2-yl]oxy}oxan-2-yl]oxy}-5-hydroxy-2-(4-hydroxyphenyl)-3,4-dihydro-2H-1-benzopyran-4-one | C_27_H_32_O_14_ | 580.1800 |  |  |  |  |

**Supplementary Table S2** Illness perceptions of patients with high, medium, and low adherence to treatment in two groups

| **Dimensions of Illness Perceptions** | **Experimental group** | | |  | **Control group** | | |  |
| --- | --- | --- | --- | --- | --- | --- | --- | --- |
|  | **Ranking of adherence to treatment** | | | ***p*-*value*** | **Ranking of adherence to treatment** | | | ***p*-*value*** |
|  | **Low**  **(n = 10)** | **Medium**  **(n = 30)** | **High**  **(n = 12)** |  | **Low**  **(n = 30)** | **Medium**  **(n = 18)** | **High**  **(n = 3)** |  |
| Consequences | 3.5 (1-10) | 4 (1-8) | 4.5 (1-9) | 0.647 | 4.5 (1-8) | 4.5 (0-7) | 7 (5-8) | 0.133 |
| Timeline | 4.5 (1-10) | 5 (2-10) | 5 (2-9) | 0.859 | 7 (4-10) | 6 (4-10) | 5 (3-10) | 0.339 |
| Personal control | 6.5 (4-8) | 4 (2-7) | 5 (4-9) | 0.000 | 5.5 (2-9) | 5 (3-8) | 6 (4-6) | 0.607 |
| Treatment control | 5.5 (2-7) | 4 (0-6) | 5 (0-7) | 0.134 | 5 (1-8) | 5 (3-10) | 6 (5-7) | 0.452 |
| Severity of symptoms | 4 (2-5) | 4 (1-8) | 5 (2-7) | 0.898 | 4 (3-9) | 4.5 (2-7) | 5 (3-7) | 0.808 |
| Concerns | 4 (2-9) | 6 (3-8) | 6 (0-8) | 0.106 | 5 (1-10) | 6 (1-10) | 4 (3-7) | 0.231 |
| Understanding | 6 (0-8) | 5 (2-8) | 5 (1-10) | 0.061 | 6 (0-10) | 5 (3-6) | 3 (2-6) | 0.059 |
| Emotional representation | 3 (1-10) | 1 (0-8) | 2 (0-5) | 0.168 | 2 (0-8) | 2 (0-6) | 2 (1-10) | 0.917 |
| BIPQ | 38.5 ± 7.663 | 34.33 ± 8.049 | 35.75 ± 5.083 | 0.311 | 38.9 ± 6.53 | 37.72 ± 6.614 | 41.67 ± 8.145 | 0.605 |

**Supplementary Table S3** Blood routine and blood biochemical indexes

| **Index** | **V1** | | ***p*-*value*** | **V3** | | ***p*-*value*** |
| --- | --- | --- | --- | --- | --- | --- |
|  | **Experimental** | **Control** |  | **Experimental** | **Control** |  |
| WBC (10^9^/L) | 5.80 ± 1.39 | 5.85 ± 1.59 | 0.933 | 5.83 ± 1.58 | 5.73 ± 1.75 | 0.759 |
| RBG (10^12^/L) | 4.36 ± 0.44 | 4.62 ± 0.37 | 0.931 | 4.55 ± 0.41 | 4.54 ± 0.43 | 0.902 |
| PLT (10^9^/L) | 256.49 ± 97.45 | 243.12 ± 59.46 | 0.404 | 256.38 ± 93.07 | 245.47 ± 63.67 | 0.490 |
| HGB (g/L) | 139.27 ± 13.97 | 137.20 ± 16.37 | 0.491 | 138.06 ± 14.78 | 134.71 ± 18.07 | 0.305 |
| TP (g/L) | 74.29 ± 4.40 | 74.96 ± 3.86 | 0.413 | 73.53 ± 5.42 | 74.07 ± 4.16 | 0.576 |
| ALB (g/L) | 45.56 ± 2.48 | 45.60 ± 2.41 | 0.933 | 45.44 ± 2.67 | 45.76 ± 4.57 | 0.663 |
| ALT (U/L) | 23.89 ± 19.83 | 18.50 ± 11.58 | 0.095 | 23.54 ± 18.42 | 20.70 ± 15.58 | 0.401 |
| AST (U/L) | 21.49 ± 9.26 | 20.15 ± 6.66 | 0.401 | 20.76 ± 7.99 | 21.92 ± 9.48 | 0.506 |
| BUN (mmol/L) | 5.24 ± 1.28 | 4.80 ± 1.67 | 0.137 | 5.18 ± 1.34 | 4.67 ± 1.19 | 0.043 |
| SCR (μmol/L) | 68.89 ± 16.57 | 62.90 ± 13.91 | 0.050 | 67.05 ± 18.96 | 62.29 ± 13.98 | 0.150 |

**Note:** WBC, white blood cell; RBG, red blood cell; PLT, platelet; HGB, hemoglobin; TP, total protein; ALB, albumin; ALT, alaninetransaminase; AST, aspartate transaminase; BUN, blood urea nitrogen; SCR, serum creatinine

# Reference

1. Montone CM, Moneta BG, Aita SE, Aulenta F, Cavaliere C, Cerrato A, et al. Untargeted analysis of contaminants in river water samples: Comparison between two different sorbents for solid-phase extraction followed by liquid chromatography-high-resolution mass spectrometry determination. Microchemical Journal. 2022;172:106979-.

2. Wang D, Fu Z, Xing Y, Tan Y, Han L, Yu H, et al. Rapid identification of chemical composition and metabolites of Pingxiao Capsule in vivo using molecular networking and untargeted data-dependent tandem mass spectrometry. Biomed Chromatogr. 2020;34(9):e4882.

3. Xiaohui F, Yi W, Yiyu C. LC/MS fingerprinting of Shenmai injection: a novel approach to quality control of herbal medicines. J Pharm Biomed Anal. 2006;40(3):591-7.

4. Geng P, Harnly JM, Chen P. Differentiation of bread made with whole grain and refined wheat (T. aestivum) flour using LC/MS-based chromatographic fingerprinting and chemometric approaches. Journal of Food Composition & Analysis. 2016;47:92-100.

5. Cao Y, Chai C, Chang A, Xu X, Song Q, Liu W, et al. Optimal collision energy is an eligible molecular descriptor to boost structural annotation: An application for chlorogenic acid derivatives-focused chemical profiling. J Chromatogr A. 2020;1609:460515.

6. Liu JJ, Cheng Y, Shao YY, Chang ZP, Guo YT, Feng XJ, et al. Comparative pharmacokinetics and metabolites study of seven major bioactive components of Shaoyao-Gancao decoction in normal and polycystic ovary syndrome rats by ultra high pressure liquid chromatography with tandem mass spectrometry. J Sep Sci. 2019;42(15):2534-49.

7. Kwon M, Ji HK, Goo SH, Nam SJ, Kang YJ, Lee E, et al. Involvement of intestinal efflux and metabolic instability in the pharmacokinetics of platycodin D in rats. Drug Metab Pharmacokinet. 2017;32(5):248-54.

8. Jia X, Liu J, Shi B, Liang Q, Gao J, Feng G, et al. Screening Bioactive Compounds of Siraitia grosvenorii by Immobilized β(2)-Adrenergic Receptor Chromatography and Druggability Evaluation. Front Pharmacol. 2019;10:915.
